# Boar Sperm Motility Assessment Using Computer-Assisted Sperm Analysis: Current Practices, Limitations, and Methodological Challenges

**DOI:** 10.3390/ani15030305

**Published:** 2025-01-22

**Authors:** Lenka Hackerova, Aneta Pilsova, Zuzana Pilsova, Natalie Zelenkova, Pavla Tymich Hegrova, Barbora Klusackova, Eva Chmelikova, Marketa Sedmikova, Ondrej Simonik, Pavla Postlerova

**Affiliations:** 1 Department of Veterinary Sciences, Faculty of Agrobiology, Food, and Natural Resources, Czech University of Life Sciences Prague, 16500 Prague, Czech Republic; hackerova@af.czu.cz (L.H.); pilsova@af.czu.cz (A.P.); pilsovaz@af.czu.cz (Z.P.); zelenkovan@af.czu.cz (N.Z.); pokornapavla@af.czu.cz (P.T.H.); klusackovab@af.czu.cz (B.K.); chmelikova@af.czu.cz (E.C.); sedmikova@af.czu.cz (M.S.); 2 Laboratory of Reproductive Biology, Institute of Biotechnology of the Czech Academy of Sciences, BIOCEV, 25250 Vestec, Czech Republic; ondrej.simonik@ibt.cas.cz

**Keywords:** ejaculate quality, in vitro analysis, hyperactivation, kinematic parameters, reproductive physiology, male fertility, veterinary science

## Abstract

Spermatozoa are highly specialized male cells that are characterized by a unique ability to move, which is a critical factor for successful fertilization. The relative simplicity of motility assessment, especially in livestock, has made it a widely used parameter for evaluating ejaculate quality or cryopreserved semen in the clinical field, and an advanced tool in reproductive physiology and toxicology research. Technological advances in image analysis and computational methods have substantially increased its accuracy through the use of computer-assisted sperm analysis (CASA) to minimize subjective bias in motility assessments. Nevertheless, this more objective method still presents some significant challenges, including variability in the sample preparation, imaging conditions, and analytical parameters. These issues contribute to inconsistency and impair the reproducibility and comparability of data between laboratories. The implementation of standardized protocols, combined with comprehensive training and rigorous evaluation, can serve to mitigate some of the emerging inconsistencies. In addition, the in vitro conditions under which CASA analyses are performed often differ significantly from the natural environment of the female reproductive tract in vivo. This review discusses the methodologies, critical issues, and limitations of sperm motility analyses using CASA, with a particular focus on the boar as an important agricultural and biomedical model species in which this system is widely used.

## Simple Summary

Spermatozoa are specialized cells with the very unique ability to move, which is called motility. This is one of the main properties of sperm and is essential for in vivo fertilization. There are two basic ways to evaluate this property: subjectively, and more objectively using Computer-Assisted Sperm Analysis (CASA). CASA is currently being used more and more in clinical practice as well as in research, where it is already standard. However, in addition to its many advantages, this type of sperm motility analysis can be hampered by many factors that can affect its results. Our review provides a comprehensive view of this issue with an emphasis on the latest findings, the standardization of the preanalytical part of CASA, statistical processing, and biological value. We provide a valuable summary of this issue that is suitable for both the field of research in reproductive biology and for clinical veterinary practice.

## 1. Introduction

Spermatozoa are specialized male cells that have a unique ability—motility. This allows them to achieve their primary goal, which is to fertilize the oocyte. Although sperm motility alone is not a sufficient criterion for fertilizing ability, it is clear that the acquisition, maintenance, and modulation of motility are among the universally recognized aspects of male fertility potential [1]. As it is relatively easy to assess [2], sperm motility in livestock is not only used as a major factor in rapid ejaculate selection and quality assessments of preserved sperm, but is also used as an important research tool to improve male reproduction and knowledge of reproductive toxicology and sperm physiology [3]. With the gradual improvements in image analysis and computing technologies, great progress has been made in the development of automated sperm motility assessments, which are known as computer-assisted sperm analysis (CASA), with the primary intention of eliminating bias in results obtained via subjective assessment [4]. Despite these technological advances, which allow for large amounts of data on a wide range of sperm characteristics to be obtained within minutes, the professional community continues to face certain difficulties and limitations that limit the potential of computer-generated data [3]. During analysis, there are a large number of variables in the methodology, such as the preparation and handling of the sample (dilution medium, temperature, and dilution factor), the random selection of which part of the field is analyzed, the selection of the correct optics, the frequency of imaging, and the form of analysis itself, which may not be consistent [5]. Even though the standardization of assessments is considered a major advantage of the CASA system, the data obtained usually remain highly laboratory-specific for the above reasons [3]. The implementation of detailed standard operating procedures, as well as thorough training and the regular evaluation of laboratory personnel, could significantly reduce potential intra-laboratory variation [6,7], but these methods are unlikely to eliminate deficiencies in interlaboratory comparability unless identical equipment and standard operating procedures are used. Moreover, the analysis usually takes place under conditions that are significantly different from the in vivo environment of the female reproductive tract [3]. All these aspects indicate that the data generated by research laboratories and laboratories in insemination centers are often not consistent, and are therefore practically incomparable. The aim of this review is to provide a comprehensive overview of the approaches, main pitfalls, and problems in sperm motility analyses using the CASA system, especially in boars, which are model animals in farms and the biomedical field, in which evaluations of sperm motility are frequently carried out using this system.

## 2. Formation and Regulation of Sperm Motility

The development of sperm motility is highly dependent not only on the correct development of the flagellum, but also on the sperm maturation process in the epididymis [8], where species-specific morphological, biochemical, and physiological changes occur [9]. As spermatozoa enter the epididymal tubule in a transcriptionally inactive state with limited biosynthesis capacity [10], any changes must be driven by the dynamic intraluminal environment to which spermatozoa are exposed during passage through the epididymis [11]. Important components of this environment are calcium (Ca^2+^) and bicarbonate (HCO_3_^−^) ions, which, through the regulation of soluble adenylyl cyclase (sAC), are significantly involved in the activation and control of sperm motility [12].

The key role of sAC is the generation of cyclic adenosine monophosphate (cAMP), an increased production of which leads to the progressive development of the cAMP/PKA (protein kinase A) signaling pathway, which is associated with the potential activation of motility and the ability of sperm to undergo the capacitation process necessary to acquire the ability to fertilize [13,14,15,16]. The activation of cytosolic cAMP-dependent protein kinases, including PKA, is generally reported to increase the phosphorylation of intrasperm phosphoproteins that can regulate flagellar motility [16]. These target proteins also include non-enzymatic proteins of the A-kinase anchoring protein (AKAP) family, which are localized to the flagellum of mammalian spermatozoa [17]. AKAPs restrict PKA activity to specific microdomains depending on the regulation of the PKA-AKAP interaction. Most AKAPs also anchor other signaling enzymes, such as cyclic nucleotide phosphodiesterases, which degrade cAMP and allow PKA activation to be locally regulated in terms of amplitude and duration [18]. Specifically, AKAP4s appear to be essential for coordinating PKA signal transduction to motor proteins such as dyneins, thereby promoting the sliding of microtubule doublets through the axoneme [19]. It is the phosphorylation of dyneins and other proteins associated with the axonemal cytoskeleton that is essential for the regulation and generation of flagellar movement [20].

In addition to the cAMP/PKA pathway, the Ca^2+^/calmodulin pathway is also involved in protein phosphorylation and motility regulation, which regulates the activity of many enzymes, including Ca^2+^/calmodulin-dependent protein kinases (CaM kinases) [12,21,22]. Despite the general assumption that the induction of sperm motility in the epididymis is associated with a gradual transition from phosphatase activities to kinase activities, and therefore with gradual protein phosphorylation [23], the exact mechanisms are not fully clarified, even after decades of research. In addition, existing interspecies differences greatly complicate the entire clarification [20]. More detailed information on sperm maturation and motility activation is available in the review by Dacheux and Dacheux [12].

**Figure 1:**
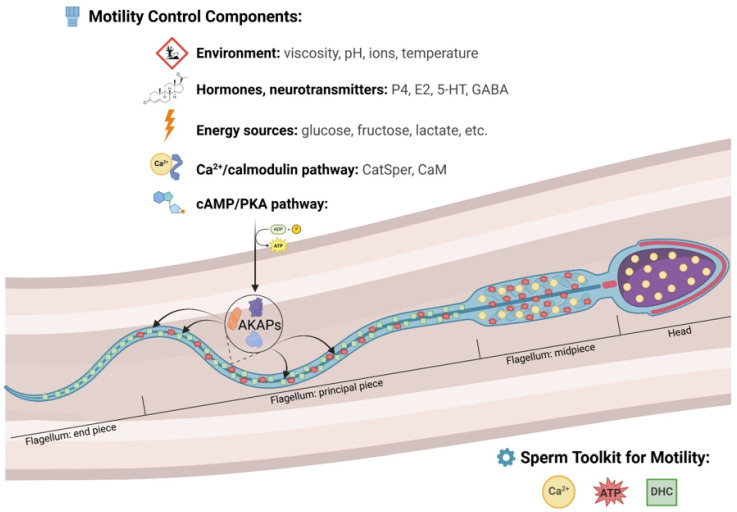
Components of motility control. In addition to the environment, hormones, neurotransmitters, and the availability of energy sources, two main pathways are involved in the control of motility—the calcium (Ca^2+^)/calmodulin pathway and the cyclic adenosine monophosphate (cAMP)-dependent protein kinase A (PKA) pathway. Legend: P4, progesterone; E2, estradiol; 5-HT, 5-hydroxytryptamine; GABA, γ-aminobutyric acid; CaM, calmodulin-dependent protein kinases; CatSper, cation channels of sperm; cAMP, cyclic adenosine monophosphate; PKA, protein kinase A; P, phosphate; ADP, adenosine diphosphate; ATP, adenosine triphosphate; Ca^2+^, calcium ions; DHC, dynein heavy chains. Created with BioRender.com.

Thus, it is clear that sperm motility is a complex process dependent on many external and internal factors (schematically shown in Figure 1) associated with intracellular signaling pathways and post-translational modifications [24]. It is complex cellular signaling pathways initiated by extrinsic and intrinsic factors that alter and determine flagellar movement and the sperm swimming pattern, such that spermatozoa exhibit different swimming patterns in the epididymis, seminal plasma, cervical mucus, and oviduct [25,26]. Two basic patterns of motility have been characterized in spermatozoa: (1) active and (2) hyperactive motility [27]. While active motility, characterized by low amplitude, symmetric flagellar beat, and nearly straight trajectory, can be observed in freshly ejaculated spermatozoa moving in a low-viscosity fluid [28,29], the hyperactivation of motility occurs primarily at the site of fertilization. Even though the movement of hyperactivated spermatozoa varies under different physical conditions and in different species, in general, it can be said that it is a less progressive, highly energetic movement by the asymmetric oscillation of the flagellum with an irregular trajectory [22,30,31,32], which is accompanied by other physiological changes necessary for the acquisition of fertilization ability, collectively known as capacitation [33]. This type of movement increases the sperm’s ability to detach from the oviductal epithelium [34] and penetrate the viscous fluids of the oviduct as well as the matrix of the *Cumulus oophorus*. It is thus a key physiological process necessary for the fertilization of the oocyte [34,35,36], which is, however, highly energy-demanding. If hyperactivation is initiated too early, there is a risk of depleting sperm energy stores before these cells reach the site of fertilization [37,38]. In vivo, this process appears to be regulated by the balance between progesterone (P4) and 17β-estradiol, whose concentrations change during the female’s estrous cycle [39,40,41]. While progesterone acts as an inducer of sperm motility hyperactivation, 17β-estradiol suppresses progesterone-enhanced hyperactivation through tyrosine phosphorylation, inhibiting estrogen receptors [39]. In addition to these two hormones, other specific molecules of the oviductal and follicular fluid, such as melatonin, serotonin (5-hydroxytryptamine, 5-HT), and γ-aminobutyric acid (GABA), are likely to be involved in the timing of the process [39,40]. However, it seems that the effect of some molecules can be species-specific, as has been demonstrated, for example, with GABA. It acts as an inducer of the acrosome reaction and hyperactivation in human, ram, and rat spermatozoa [42,43,44,45], but as a suppressor of hyperactivation in hamster sperm [46]. In pigs, no significant effect of GABA on sperm motility was recorded [47].

The occurrence of sperm hyperactivation requires two main changes, which include “antisymmetrization of the flagellar beat” and “increase in its amplitude”. Numerous studies have shown that the most important initiator of these changes is Ca^2+^ ions, whose rapid increase is induced by influx from the extracellular space and recruitment from internal stores [17]. From the extracellular environment, Ca^2+^ ions enter the sperm through specific cation channels (CatSper) [48] located in the membrane of the main compartment of the flagellum [49]. The internal stores of Ca^2+^ ions in mammalian sperm are mediated by two functional intracellular deposits [50], which in boar sperm are located throughout the head and in the middle part of the flagellum [51]. Chang and Suarez [52] demonstrated two patterns of hyperactivation in mouse sperm, anti-hook and pro-hook, depending on the Ca^2+^ ion source, which was later confirmed in boar and bull spermatozoa as full-type and nonfull-type, equivalent to mouse patterns [53,54,55]. Full-type hyperactivation, which is dependent on extracellular Ca^2+^ on the order of mM, is characterized by completely asymmetric flagellum beats with greater amplitude in the middle and main compartments. On the contrary, in nonfull-type hyperactivation, which is triggered by the release of Ca^2+^ from intracellular stores, asymmetric and large beats are mainly limited to the distal part of the middle section of the flagellum [53]. Ejaculated and in vivo capacitated mouse sperm mainly exhibit asymmetric beats in the oviduct of females with a larger amplitude in the whole parts of the middle and main section of the flagellum, i.e., the anti-hook type equivalent of the full-type hyperactivation of boar sperm [17,56]. These findings suggest that full-type hyperactivation is the physiological (in vivo) pattern of boar sperm [17].

Although hyperactivation occurs during the residence of spermatozoa in the female reproductive tract [38,57], this process can be induced in vitro in human and mouse sperm using a medium that mimics the conditions of the female reproductive tract [58]. However, it appears that the induction of hyperactivation by simple incubation in a capacitation-promoting medium can be difficult in boar spermatozoa [17]. One explanation is that these media could have less potential to activate cAMP/protein phosphorylation-dependent signaling cascades, which are coupled to Ca^2+^-dependent signaling cascades, leading to flagellar hyperactivation [17,59]. Another explanation suggests the presence of strong suppressors (e.g., calyculin-sensitive protein phosphatase) for boar sperm hyperactivation [54]. However, it has been shown that hyperactivation in non-capacitated boar spermatozoa can be easily induced in vitro by treatment with intracellular Ca^2+^ level elevators (e.g., A23187 or thimerosal), although the activation of intracellular cAMP/PKA signaling cascades can be skipped in these spermatozoa [17].

Although there are still many questions surrounding hyperactivation, it can be said that it is a reversible process requiring a permanent increase in intracellular Ca^2+^ ions. As a result of their changing level, a so-called biphasic behavior is observed in the sperm of some animal species, i.e., an alternation of active and hyperactive movement [31,60,61]. To study, evaluate, detect, and identify the percentage of activated and hyperactivated spermatozoa in a sample, it is necessary to use computer-assisted sperm analysis (CASA) because identifying hyperactivated spermatozoa with the naked eye is very difficult [62]. Furthermore, the exact movement pattern of hyperactivated sperm is difficult to define because it varies among species depending on the thickness and length of the flagellum and the characteristics of the physical environment (discussed below, see Section 4.2) in which the male gametes are currently moving [31].

## 3. Computer-Assisted Sperm Analysis (CASA)

Despite mammalian spermatozoa being the most diverse cell type, CASA is a great tool that has the ability to provide a rapid and objective assessment of sperm functional aspects based on optical microscopy and 2D video micrography [63,64]. The first commercial CASA system launched in the second half of the 1980s was the then-well-known CellSoft system, with a frequency of 30 frames per second and no verification functions [4,65]. Commercial use of the fully automated CASA system in domestic animals was introduced in 1985, and a large number of more sophisticated systems with frame rates of 50 and 60 Hz came onto the market, mainly in the early 1990s [66]. Although this represented a breakthrough for the quantification of sperm motility, which is thus a traditional parameter and a core function of the CASA system [67], these were mostly black boxes with little real-world validation and were viewed with some skepticism [68]. However, over the past 20 years, advances in hardware and software have revolutionized CASA systems, whereby most systems now provide a high level of quality control and verification [64,69]. The latest generations of CASA systems also include improved algorithms for the evaluation of sperm characteristics such as DNA fragmentation, morphology, viability, acrosome reaction, and semi-quantitatively, hypoosmotic pressure [64]. However, with the development of algorithms for the evaluation of new characteristics, the conventional CASA terminology began to be inadequate, as the CASA acronym itself is uninformative and does not say anything about which aspect of spermatozoa is being analyzed. Although the term CASA is now generally used mainly in connection with sperm kinematics, the first studies used this general term to cover all aspects. Currently, more appropriate names indicating what is actually being assessed are adopted, i.e., CASA-Conc (concentration), CASA-Mot (motility and/or kinematics), CASA-Morph (morphology and/or morphometry), CASA-DNA (DNA), which can also be expanded by other specifications if necessary, such as the use of a fluorescent dye for morphology (Casa-Morph-F) [70].

Currently, the most commonly used systems in the veterinary field, in order of estimated market share, are as follows: IVOS II/CEROS II (Hamilton Thorne, Beverly, MA, USA), AndroVision and Androscope (Minitube, Tiefenbach, Germany), SCA (Microptic, Barcelona, Spain—now part of Hamilton Thorne), ISAS (Arquimea, Madrid, Spain), Magavion (Magapor, Zaragoza, Spain), iSperm (Aidmics, Taipei, Taiwan), OCSA (Kubus, Madrid, Spain), SQA (Medical Electronic Systems, Encino, CA, USA), AI Station (SpermTech, Valencia, Spain), and occasionally used in academia, Open CASA on ImageJ (https://imagej.net/ij/). CASA systems with specific boar sperm CASA-Mot programs are limited to IVOS II, AndroVision, CEROS II, Magavision, SCA, OCSA, and iSperm (partially reviewed by Boe-Hansen and Satake [67]).

The main components of the CASA system include a microscope equipped with a heated stage, an optical device with negative phase contrast, and an attached video camera and specific software that receives the camera signal and enables the calculation of the obtained data according to the type of analysis [71,72]. Most CASA systems work by converting the captured image of the microscope field into a digital image using a highly negative phase contrast image that shows white sperm heads against a dark background. The computer recognizes the object as a sperm head according to its expected minimum and maximum size, depending on the animal species. After identifying the head of the sperm, the computer determines its position (x, y) according to the so-called reference point, which is determined either by the center of the head or its brightest point. When all identified sperm heads in one field are computerized, the next field is analyzed. The radius in which the computer searches for the image of the head is given by the assumed maximum distance [73] that the sperm can move in a certain time interval [74]. Subsequently, the sperm coordinates (x, y) are calculated, and the next field is analyzed until the scan is complete. For each spermatozoon, the reference point trajectory is reconstructed, and a series of kinematic values is calculated [73]. The first and last five track points are discarded from the analysis to avoid track initiation and track termination artifacts that confound motility estimates [75].

### 3.1. Kinematic Parameters of Sperm Motility

Commercially used CASA systems can determine at least seven kinematic parameters of sperm motility (van der Horst 2020) [66], whose data are obtained on the basis of a set of sperm reference point position measurements associated with the entire track history and which are available as a database file intended for subsequent processing and analysis [75]. In AI centers, one of the basic kinematic parameters is primarily determined, namely either progressive motility (i.e., the percentage of progressively motile sperm), which depends on structural and functional cellular integrity and is influenced by the environment surrounding the sperm, or total motility (i.e., the percentage of motile sperm). It is the total motility that is generally determined for the assessment of boar spermatozoa [3]. More specific kinematic parameters include three sperm movement speed parameters—curvilinear velocity (VCL), straight-line velocity (VSL), average path velocity (VAP); three speed ratio parameters—linearity (LIN), straightness (STR), wobble (WOB); and three parameters reflecting sperm wobble characteristics—amplitude of lateral head displacement (ALH), beat cross frequency (BCF) and mean angular displacement (MAD) [74]. VCL (µm/s) is measured by summing the distance between the sperm head positions in each frame and dividing the result by the elapsed time [76]. VCL is usually the highest of the three velocity parameters [77]. VSL (µm/s) is the distance between the first and last points on the sperm track, divided by the elapsed time. VAP (µm/s) is determined by smoothing the sperm head positions in a running average. The resultant path length is determined and divided by the elapsed time [76]. If the trajectory of sperm movement is very regular and linear, VAP is almost identical to VSL. However, for the movement path of irregular sperm, VAP is much higher than VSL [74]. LIN (percent) is the ratio VSL/VCL in percent and is a measure of track direction, STR (percent) is the ratio VSL/VAP in percent and is a measure of track compactness, WOB (percent) is the ratio VAP/VCL. ALH (µm) is the maximum value of the approximately sinusoidal oscillation of the sperm head about the track. It is measured as the maximum distance between the actual sperm position and the corresponding average sperm position for all points over the track. BCF (Hz) is the frequency with which the sperm head crosses the average path line during acquisition [76]. MAD (degrees) is the time-averaged absolute values of the instantaneous angle of rotation of the sperm head along its curvilinear trajectory [77,78]. Additionally, the Hamilton Thorne CASA II system defines and measures parameters such as distance straight line (DSL), distance curvilinear (DCL), and distance average path (DAP). DSL (microns) refers to the distance traveled by the sperm along the VSL path. DCL (microns) represents the total distance covered by the sperm along its curvilinear path, while DAP (microns) denotes the distance traveled by the sperm along the VAP path [76]. As ALH and VAP are calculated based on algorithms that differ between instruments, values might not be comparable between CASA systems [77]. This can also lead to significant differences in WOB, STR, and BCF [74].

Additional new parameters and visualizations that can help better define sperm function and quality are coming with the development of specific and new approaches that complement CASA. The measurement of several new quantitative parameters, such as the frequency of the flagellar beat, the energy expenditure (in watts) expended by each spermatozoon, or the curvature of the flagellar wave to determine the length of the arc (the actual length of the trace), is being made possible by the program FAST [66,79]. This, together with a newly developing three-dimensional (3D) sperm analysis system, could provide new insights into male germ cell biology and fertility assessment [66]. The three-dimensional (3D) system analyzes sperm tracks in three dimensions (X, Y, and Z axes), which contributes to a better simulation of in vivo conditions, since the sperm of most animal species swim in a spherical helix [66,68].

### 3.2. CASA Data Analysis

The sperm motility data obtained through the CASA system were previously evaluated mainly using simple statistics such as the mean of the variables [80]. It was on the basis of the averages that threshold values of kinematic parameters were determined, according to which active and hyperactive spermatozoa began to be characterized. Receiver operating characteristic (ROC) curve analysis of sperm in the Schmidt and Kamp [81] study suggested that ALH alone is sufficient to distinguish between hyperactive and non-hyperactive sperm. However, at the same time, the authors stated that this monoparametric criterion does not take into account the increased curvilinear velocity generated by the deeper bend of the flagella, nor the reduced linearity due to their asymmetric strokes [82]. Mortimer and De Jonge [62] also disagreed with the monoparametric criterion, which drew attention to the complexity of the trajectories and stated that there is no single kinematic parameter that would reliably define hyperactivated motility. However, the HT CASA II system employs the fractal dimension (FDM) algorithm as a specialized method to identify hyperactivated spermatozoa. The FDM for a sperm track is calculated using the formula FDM = log(n)/[log(n) + log (d/L)], where d represents the maximum DSL for the track, L is the total DCL, and n is the number of points in the track minus one. A track is classified as fractal if the FDM exceeds 1.3. FDM was originally designed for analyzing human sperm but has since been adapted for use with other species [73,83,84,85].

Studies have found that mean (threshold) values of kinematic parameters differ within individual species, reflecting species-specific patterns of sperm movement. Although there are some differences in absolute values even between individuals of the same species (see Table 1), a general observation in boar sperm hyperactivation seems to be a decrease in VSL and LIN and an increase in VCL and ALH [81] leading to a highly energetic movement that makes little forward progress. However, this phenomenon is observed in standard media, where an uncontrolled increase in sperm head amplitude occurs due to the lack of viscous damping of active flagellar bending. At physiological viscosity, these spermatozoa would swim forward very efficiently because the viscous forces would “cancel out” the deflection, as indicated by data obtained in mouse sperm [35]. Therefore, the observation of hyperactivation at the viscosity of the physiological medium requires a reinterpretation of the data obtained.

However, it has been shown that this simple approach based on the mean values of variables does not take advantage of the amount of data available, and therefore, some relevant information cannot be evaluated at all [89]. The need to obtain these results provided the impetus for the development of multivariate analysis, especially descriptive approaches (e.g., PCA principal component analysis) or clustering (e.g., data clustering) [80]. Thanks to this, various authors found that the actual distribution of sperm is not uniform, but structured into different subpopulations defined by their mobility and kinematic characteristics [90,91,92,93]. Different studies usually report the presence of three to four sperm subpopulations, which are characterized by specific values of velocity and linearity [94,95]. Although the issue of the meaning and origin of sperm subpopulations is still too complex to be fully understood [80], some authors believe that the interpretation of CASA results based purely on mean values is currently already completely inadequate [95].

Although the analysis of sperm subpopulations based on CASA output has been a highly discussed topic [95], there are still no standardized procedures for evaluating the subpopulations of (not only) boar ejaculates [3,7]. This is due to the fact that there are a large number of variables involved in the whole process, which causes differences between studies in the absolute values of kinematic parameters characterizing individual sperm subpopulations.

## 4. Aspects Affecting Sperm Motility and Data Obtained During CASA

CASA systems were developed as a promising alternative to the conventional subjective evaluation of light microscopy. Currently, this type of analysis is very user-friendly and is able to provide a huge amount of accurate and objective data in a short period of time [4]. However, due to a number of factors influencing CASA measurements [96], the data obtained in most cases remain highly laboratory-specific [3]. In recent years, the importance of developing and publishing detailed methodological protocols for the effective use of CASA systems in the analysis of semen of individual animal species has been emphasized, as well as the need for standardization in CASA evaluation to reduce interlaboratory variability, which is a persistent challenge due to different protocols, extender types and hardware configurations across artificial insemination (AI) centers [6,97]. However, studies are beginning to emerge that present standardized CASA procedures and settings for individual animal species, which facilitate replicable methodologies and have the potential to reduce the aforementioned interlaboratory variability [97,98]. So, what factors influence the data obtained and should be considered when creating standardized protocols? These factors include the choice and use of analysis slides with chambers, the settings of the semen analysis devices, the type of software used, the analysis time, the number of frames per second during the analysis, the generating trajectories and velocity calculation algorithms, the operator variability, the sample preparation including semen analysis medium, semen dilution (overview in the work of Camus et al. [99]), the age of the individual [100,101], and the breed within the animal species [102,103,104].

### 4.1. Type of Counting Chamber

Sperm must be placed in a disposable or reusable chamber for analysis by the CASA system [93,105]. The counting chamber is considered to be one of the two most important factors influencing the concentration, motility, and kinematic parameters of sperm motility [105,106]. Cell filling can take place in two ways based on general physical principles, either (1) by capillary action in most disposable chambers (e.g., Cell-Vu^®^ and Leja^®^) when the sample is placed on the inlet port of the chamber (so-called Capillary-load) or (2) by moving the droplets in reusable chambers (e.g., Makler^®^), where the sample is placed directly on the glass surface and covered with a coverslip (so-called Top-load [107,108,109]). In addition, the chambers also differ in their depth, which is the distance between the ceiling and the floor of the analysis chamber [76]. Various chamber depths are available on the market, including 10 μm (e.g., Makler^®^ and Leja^®^), 12 μm (e.g., Leja^®^), 20 μm (e.g., Cell-Vu^®^, Leja^®^, MicroCell™), and 100 μm (e.g., Leja^®^). Therefore, three primary variables are related to the effect of the counting chamber, namely the construction of the chamber, its depth, and the filling method [105].

According to available studies (reviewed by Bompart et al. [105]), different chamber constructions affect the final distribution of sperm inside the chamber and their motility properties in many animal species, including boars [110]. A study by Basiour et al. [96] performed on boar spermatozoa showed that Makler chambers, compared to Leja chambers, are associated with higher results of total motility, progressive motility, velocity parameters (VCL, VSL, VAP), STR, and hyperactivity, indicating a systematic effect. That the motility and kinematic parameters observed in capillary chambers exhibit lower values than those observed in chambers filled by droplet transfer has been demonstrated through various CASA systems and in other animal species, including bulls [106,111], rams [112], and stallions [113]. Thus, the principle of sample distribution appears to be more important than species differences or the actual brand of counting chambers or CASA systems [106]. A plausible explanation could be that capillary action can damage the sperm tail and disrupt sperm motility due to the resulting fluid flow [106,112,114].

However, there is a study by Gączarzewicz et al. [115] on boar spermatozoa, which, using the same CASA system, produced the opposite results to the study by Basiour et al. [96]. While in the deeper Leja chamber (20), sperm were observed to move in irregular trajectories with high energy (higher VCL, VAP, ALH, and BCF) and lower progressivity (lower STR and LIN); in the Makler chamber (10), sperm moved less energetically (lower VCL, VAP, ALH, and BCF) along more rectilinear trajectories (higher STR and LIN). The authors explain these results by the fact that the greater depth of the Leja chamber allows sperm to move more freely [115] since in a shallower chamber, the natural (three-dimensional) movement of sperm is probably suppressed by the restriction of the movement of the flagellum in one of the dimensions [4,116]. In addition, sperm velocity and movement can also be affected by proximity to the chamber surface, as sperm tend to adhere to it, potentially depending on surface tension [4,114]. Adherence to the chamber surface was also observed in boar spermatozoa, which exhibited flagellar movement but were in fact static [93].

The importance of counting chamber depth as one of the main factors influencing sperm dynamics [74] was also the subject of a study by Lannou et al. [117]. They demonstrated that sperm dynamics, especially in connection with the hyperactivation of motility, need to be analyzed in a chamber with a depth of at least 20 μm. Mortimer [73] even believes that a 20 μm depth of the counting chamber may still limit the movement of the flagellum. Therefore, it is necessary to use a chamber with a depth of at least 30 μm to analyze the percentage of hyperactive spermatozoa. However, the limiting factor is the depth of field, i.e., the axial distance between the nearest and the farthest plane in which the sample appears to be in focus [4]. Along with the increasing depth of the counting chambers, the spermatozoa are observed at different levels, which inevitably affects the sharpness of the sperm image. If sperm are out of focus, CASA-Mot systems cannot provide any information and data for these cells [105,118]. Therefore, CASA devices are primarily used in combination with shallow chambers (for boar sperm—20 μm) to keep moving spermatozoa in the focal plane of the microscope and thus obtain a sharp image of spermatozoa when performing motility analysis [113].

However, the last decade has seen the development of new microscopy technologies based on laser light and holographic analysis, opening up a new spectrum of possibilities for the analysis of motility patterns in 3D imaging using chambers with depths > 100 μm [119,120,121,122]. A study by Soler et al. [118] performed on boar sperm used lensless Digital Holographic Microscopy (DHM) with the new CASA-Mot ISAS^®^3D-Track system with a fast frame rate (FR) of 100 fps for sperm analysis to assess the effects of chamber depth (10, 20, and 100 μm) on kinematic parameters of sperm motility. Classical probability statistics showed that when analyzed in a 100 μm deep chamber compared to a 10 and 20 μm deep chamber, the values of all sperm kinematic parameters increase, especially VSL, VAP, VCL, ALH, and BCF. These results suggest that spermatozoa exhibit significant changes in their motility patterns when they move more naturally in a 3D environment without interfering with glass surfaces. The ability to perform 3D analysis in deeper chambers is thus a prerequisite not only for the evaluation of sperm hyperactivation during their capacitation, but also for the study of interactions between sperm and oocytes in different animal species [118].

The type and shape of the imaging chamber can also affect sperm distribution and concentration, which can subsequently significantly affect the analysis of the kinematic parameters of sperm motility [87,111,123]. In chambers filled by capillary action, the fluid follows a laminar Poiseuille flow, which causes the flow velocity to be non-uniform and increase with distance from the capillary walls towards the center of the channel. The sperm suspension is thus pushed transversely to the walls, resulting in an uneven distribution of suspended particles in the sample known as the Segre–Silberberg (SS) effect [124,125]. The influence of this SS effect on the sperm distribution on the slide also depends on the sample’s viscosity. However, correction factors are available based on the time it takes to fill the chamber [126]. Studies by Rijsselaere et al. [123] and Palacín et al. [112] observed a greater sperm concentration in the outermost fields due to the SS effect, which increased sperm motility in this area. The same results were also found in a study by Del Gallego et al. [108], who observed that sperm motility assessed in capillary-filled chambers was lower in the central fields than in the outermost microscopic fields, while in droplet-filled chambers, motility values were similar in all microscopic fields.

Despite the fact that different types and depths of counting chambers are available on the market, simple microscope slides with a coverslip still remain the main choice for CASA analysis for many laboratories, mainly for economic reasons. Comparing the effect of these slides with a coverslip versus specialized slides with chambers for CASA sperm motility analysis has been the subject of a number of studies. Ratnawati and Luthfi [127] demonstrated that while there was no statistically significant difference in overall motility, progressive motility, VCL, VSL, VAP, LIN, STR, WOB, ALH, and BCF parameters between the Leja chambers and a microscope slide with a coverslip, hyperactive sperm values were significantly higher (*p* < 0.05) when using a Leja^®^ than when using a slide–coverslip. In contrast, the study by Robinson et al. [128] demonstrated that although no significant difference in progressive motility and overall motility was observed between a MicroCell™ chamber and a microscope slide, significant differences were observed in sperm kinematic parameters such as VCL, VAP, and VSL. These findings were consistent with other studies [113,114,129]. However, in the study by Palacín et al. [112], the highest variability was observed in the precision study for samples analyzed using a microscope slide and coverslip. This variability may be due to significant differences between the central and peripheral fields. When a coverslip is applied, the sperm sample drop is flattened to cover the entire area under the coverslip, which causes sperm flow and differential distribution. Sperm with different levels of motility can respond differently to such flow [130]. While dead sperm move towards the periphery of the drop due to the flow, motile sperm, on the other hand, reflexively swim upstream in response to flow stimuli and move to the center of the drop, a phenomenon known as rheotaxis [99]. After flattening the sperm suspension under the coverslip, it is therefore possible that the quantitative and qualitative distribution of sperm will not be uniform under the entire surface of the coverslip—a higher amount of dead sperm in the sample leads to their accumulation at the periphery, and conversely, motile sperm are concentrated in the center of the preparation [99,130]. In such a case, motility assessment can lead to biased results. Other factors can also affect the distribution of sperm with different motility characteristics under such a coverslip, such as too large a drop volume, with which part of the suspension will be lost under the coverslip when the coverslip is flipped. This part of the suspension can contain relatively more immotile sperm, leaving a relatively larger proportion of progressively motile sperm under the coverslip. Although this bias can possibly be avoided by using a sufficiently small suspension volume, with a volume of 7 μL under a 22 × 22 mm coverslip being recommended [130], the problems of high variability resulting from sperm flow due to random placement of coverslips cannot be easily standardized, and can only be reduced by using precalibrated slides.

An important role is also played by the time between placing the sample in the chamber and its analysis, which, according to the study by Contri et al. [111] on bull spermatozoa, mainly affects the kinematic parameters of sperm velocity (VAP, VSL, and VCL), regardless of the chamber used. For this reason, it is necessary to perform the analysis within 1 or 2 min. According to the study by Del Gallego et al. [108] on goat spermatozoa, although the elapsed time between sample application and sperm evaluation does not affect the overall speed of sperm motility, progressive sperm motility and the VSL and LIN parameters decrease with increasing time delay. Sevilla et al. [131] reported that for analysis immediately after filling, a chamber filled by drop displacement (e.g., Makler^®^) produces excellent results, while for studies of longer progressivity or studies performed several minutes after filling, a chamber filled by capillary action (e.g., Leja^®^) is particularly suitable.

From the findings above, it is clear that there is no gold standard for what type of chamber/slide to use for sperm analysis when using the CASA system. Still, the counting chamber can significantly affect the data obtained on sperm motility and concentration, and thus the accuracy and interpretation of results [111,115,132]. Camus et al. [99] addressed this gap by introducing the Motility Ratio Method, which objectively validates CASA measurements by comparing theoretical and measured motility across various chamber types and CASA systems. Their findings highlight that chamber design, particularly the slide depth and filling method, can introduce significant biases, with Leja^®^ slides exhibiting lower bias than Makler^®^ chambers or coverslip methods. Incorporating such validation methods is essential to reduce variability and establish a standardized approach for analyzing sperm motility in different species.

### 4.2. In Vitro Ejaculate Handling

**Figure 2:**
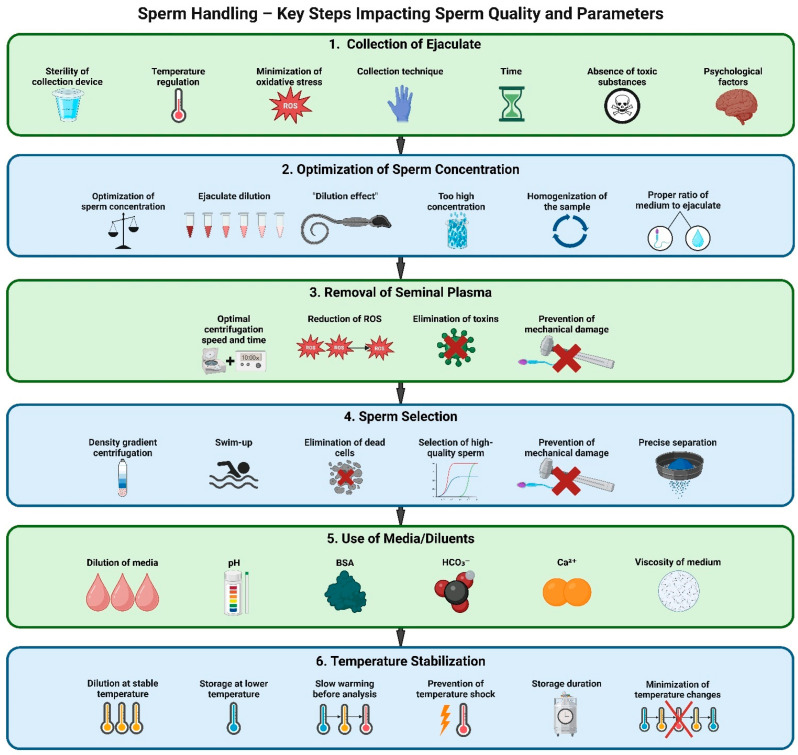
Key steps impacting quality and parameters of sperm motility. ROS, reactive oxygen species; BSA, bovine serum albumin; HCO_3_^−^, bicarbonate; Ca^2+^, calcium ions. Created with BioRender.com.

The following sections will describe the various factors that influence the CASA motility assessment of semen, which are illustrated schematically in Figure 2.

#### 4.2.1. Optimal Sperm Concentration for Accurate CASA Results

Any sample handling can affect the results obtained by CASA; therefore, it is recommended that ejaculate processing be minimized as much as possible [132]. However, boar ejaculate has a large volume of seminal plasma, and its partial or complete removal, followed by resuspension of the sperm pellets in a suitable diluent, is necessary to adjust and optimize the total sperm concentration before CASA-Mot evaluation [132,133]. A higher sperm concentration in the sample affects the progressive motility [68] and the kinematic parameters of sperm motility [134], as the number of erroneously measured data increases. This is primarily due to CASA-Mot systems’ inability to adequately detect sperm heads due to their collisions and crossings, leading to the incorrect reconstruction of individual trajectories [73,93]. On the other hand, a lower sperm concentration, i.e., a high dilution (5 and 10 × 10^6^ sperm/mL), enables a reliable reconstruction of the trajectory of each sperm. Still, the lower number of sperm detected and analyzed in the sample cannot indicate motility and its kinematic parameters [111]. Moreover, according to available studies (reviewed by Hayden et al. [135]) performed on sperm from various animal species, sperm diluted to less than 20 million sperm/mL appear to be subject to the so-called “dilution effect”, characterized by a dramatic decrease in sperm motility due to excessive sperm dilution [135]. This is also confirmed by the study of Contri et al. [111], who found that sperm with a concentration of 20 and 30 × 10^6^ sperm/mL exhibit, compared to sperm diluted to a concentration of 5 and 10 × 10^6^ sperm/mL, higher overall sperm motility and speed (VAP and VCL), but lower progressivity (BCF, STR, LIN). In addition, when categorizing sperm based on their speed in groups with lower sperm concentration (5 and 10 × 10^6^ sperm/mL), they demonstrated a significantly higher percentage of slow and, above all, static cells, probably caused by the rapid death of sperm after extensive dilution in a simple physiological medium, i.e., the above-mentioned “dilution effect” [111]. The effect of concentration on boar sperm motility was also investigated by Quirino et al. [136], who, after short-term incubation at 37–38 °C (≤30 min), demonstrated lower motility in a sample with a concentration of 16.7 × 10^6^ sperm/mL than in a sample with a concentration of 30 × 10^6^ sperm/mL. For these reasons, there is general agreement that the optimal sperm concentration required for reliable measurements and the generation of unbiased data should be between 20 and 50 × 10^6^ sperm/mL [68,111]. The range of these concentrations is also commonly used in boar sperm studies—20 × 10^6^ [81,137], 30 × 10^6^ [138], 20–30 × 10^6^ [139], 50 × 10^6^ [140,141].

#### 4.2.2. Impact of Seminal Plasma Removal on Sperm Motility and Viability

Due to its simple implementation, centrifugation is used as a conventional method for removing seminal plasma, followed by concentration adjustment [142]. The latter is also necessary in many boar sperm cryopreservation protocols [143]. The effects of centrifugation alone and the removal of seminal plasma on the motility of fresh boar spermatozoa were investigated by Bury et al. [144]. They demonstrated that centrifugation (1000× *g*, 10 min) and resuspension of the sperm pellet in a diluent without seminal plasma negatively affect the motility of boar spermatozoa, while the harmful effects develop over time and are particularly noticeable after 48 h of centrifugation. However, within a few minutes after centrifugation, there is a significant decrease in the speed parameters (VCL, VSL, VAP), even in the group of centrifuged sperm without removing the seminal plasma. A study by Melanda et al. [142] also obtained similar results on boar sperm, which showed that centrifugation (600× *g*, 10 min) negatively affects boar sperm total and progressive motility. Although there is still no precise explanation of how centrifugation causes sperm damage, it is hypothesized that the cause is a mechanical effect on the sperm membranes [145] as well as an indirect adverse effect caused by an excessive production of reactive oxygen species (ROS) [146]. A study by Iwasaki and Gagnon [147] demonstrated that sperm washing by repeated centrifugation and resuspension increased the reactive oxygen species detected by 20 to 50 times compared to a control sperm sample [147]. High levels of ROS are associated with sperm membrane damage through lipid peroxidation [148], which can alter sperm function, leading to loss of sperm motility and viability.

However, based on published studies, it appears that the effects of centrifugation on spermatozoa are species-specific [149], and that centrifugation time is more critical than g-force for inducing sperm ROS generation [150]. This also correlates with the results of the study by Carvajal et al. [143] on boar spermatozoa, which recommends using a relatively high g force combined with short centrifugation times, namely 2400× *g* for 3 min, to minimize sublethal damage to boar spermatozoa. Interestingly, the researchers did not observe any differences in the production of MDA (an indicator of lipid peroxidation) between the tested centrifugation regimes, and based on this, they assume that the mechanical effect of centrifugation is more a matter of sublethal sperm damage.

#### 4.2.3. Sperm Selection Protocols and Their Effects on CASA-Assessed Parameters

As part of ejaculate processing, in addition to classic centrifugation, other sperm selection methods are also commonly used, with sperm fractionation through density gradient centrifugation (Percoll R^©^) or the swim-up method, i.e., sperm selection based on their motility, being the most commonly used (overview of other methods in the work of Henkel and Schill [151]). Thanks to these methods, sperm subpopulations with different degrees of structural and functional differentiation and normality can be separated, which significantly improves the quality of the sperm obtained in the pellet [152,153]. In addition to selecting sperm subpopulations, these methods also assume the initiation of capacitation and the removal of seminal plasma, toxic and bioactive substances, dead sperm, and other cells, including leukocytes and bacteria [154].

Density gradients using solutions with colloidal silica particles are the gold standard method in the world of pig AI [155], as they can enrich the quality of samples in terms of morphology and motility [156]. After forming the gradient, the sperm sample is layered on the medium, and after a short centrifugation process, the ejaculate components settle in the part of the gradient that corresponds to their density [157]. Motile male gametes with a normal nucleus have a higher density (1.10 g/mL) than immature (1.09 g/mL) or non-motile (1.10 g/mL) spermatozoa, so due to the equalization of movements with the applied centrifugal force, they sediment faster and form at the bottom of the test tube pellet [158]. Conversely, leukocytes, cell debris, immotile, and abnormal sperm, which are the main sources of endogenous ROS in spermatozoa [159], form bands at higher levels [160]. An increased production of ROS results, among other things, in the peroxidation of membrane polyunsaturated fatty acids, which leads to a loss of sperm motility and membrane fluidity. Therefore, removing cells from higher-density gradient layers can improve sperm motility and help maintain membrane and chromatin integrity [159]. This was confirmed by the study of Noguchi et al. [156], who obtained a significantly higher percentage of motile boar sperm by Percoll separation than by simple centrifugation. The same conclusion was reached by other studies that documented the effect of Percoll in the selection of boar spermatozoa with higher motility [161,162,163,164]. The study by Matás et al. [165] evaluated the effects of centrifugation through three different discontinuous Percoll gradients (45/60, 60/75, and 45/90%) on individual parameters of boar sperm function, including motility. The study demonstrated that the percentage of total sperm motility was not affected by the choice of Percoll density gradient. However, progressive motility was higher in sperm selected with P60/75. All analyzed kinematic motility parameters were increased in Percoll-selected spermatozoa compared to the control group, with no differences between Percoll groups, except the VSL parameter, which was higher for P60/75 than for P45/60 and P45/90 [165]. Moreover, the motile sperm-enriched bottom layer exhibits lower ROS levels than unwashed samples, suggesting that separation is not accompanied by oxidative stress [159].

However, other studies have hypothesized that shear forces during density gradient centrifugation can lead to ROS production and oxidative stress in motile spermatozoa [166], especially in combination with the removal of antioxidants that are contained in the seminal plasma [147]. For this reason, new sperm selection methods that overcome centrifugation and the production of oxidative stress are still being developed, such as microfluidics inspired by the natural environment and selection processes in vivo [167].

#### 4.2.4. Diluents/Media Affecting Sperm Motility Characteristics

##### Media Components Modulating Sperm Motility and Kinematic Parameters

The physicochemical properties of the environment in which the sperm are dispersed can significantly affect CASA-Mot results [93,111]. As was mentioned above, before CASA-Mot evaluation, it is necessary to adjust the sperm concentration by diluting them in a complex diluent/medium or a solution of simple salts. However, no medium resembles the environment to which spermatozoa are gradually exposed within the female reproductive tract, especially in terms of the viscosity or concentration of bioactive ions or molecules [4]. In addition, dilution reduces the concentration of certain compounds in the seminal plasma, such as ions such as K^+^ [168] or plasma proteins, which are essential for maintaining sperm viability and motility. These losses must be compensated for by adjusting the composition of the medium.

Currently, several commercial diluents and media are available on the market, the basic components of which, such as buffers, energy substrates, proteins, salts, or substances inhibiting microbiological growth, can affect the motility results obtained through CASA. Although their effect may be less relevant if the motility assessment is performed immediately after dilution [93], it is clear that the choice of diluent or medium can significantly affect sperm motility characteristics [169,170]. How do components regulate and influence sperm motility characteristics, and what components do so?

##### The Role of BSA in Modulating Sperm Motility and Kinematic Parameters

A common compound added to capacitation media and diluents is bovine serum albumin (BSA). As a capacitating agent [171], it removes cholesterol from the membrane, leading to several important events, including a change in the motility pattern [172,173]. BSA also eliminates free radicals generated by oxidative stress and protects sperm membrane integrity against heat shock [174,175]. Waberski et al. [176] demonstrated that BSA reversibly stimulated sperm motility during a six-day storage test. This also correlates with the results of Zhang et al. [175], in which 4 g/L BSA supplementation significantly improved boar sperm motility during long-term storage at 17 °C compared to a control group without BSA. However, according to the study by Fu et al. [177], the effect of BSA on the motility of boar spermatozoa is not immediate, as a significant increase in total motility in the group of spermatozoa with BSA compared to the control group is only evident from the 5th day of storage. The effect of BSA on individual kinematic parameters of motility was also observed. Although BSA affects sperm kinematic parameter values immediately after addition, significant differences only occur after more prolonged incubation (more than 120 min). The increase in total and progressive motility and speed parameters of VCL, VSL, and VAP can be further supported when spermatozoa are incubated in a combination of BSA with 5 mM or 15 mM bicarbonate [171].

##### Bicarbonate Ions and Their Effects on CASA Outcomes

Bicarbonate ions (HCO_3_^−^), supplied to the media most often in the form of NaHCO_3_, are among the most important ions that induce sperm capacitation [178]. These molecules contribute to increasing membrane fluidity via the repacking of plasma membrane phospholipids [179,180] and stimulate Ca^2+^ uptake in boar spermatozoa. Both ions then promote the activation of soluble adenylyl cyclase (sAC), which is involved in the regulation of sperm motility by activating cAMP-dependent PKA [178]. However, some studies demonstrate that boar spermatozoa, probably due to their specific membrane structure, do not require exogenous bicarbonate to induce capacitation in vitro [171,181]. Bicarbonates are among the extracellular ions that can cause immediate and significant changes in sperm motility without changing the availability of energy substrates [182]. This effect occurs in boar spermatozoa within 5 min after contact with HCO_3_^−^ [183], while after 12 min [184] or 15 min [179], the effect of the ions begins to decrease again. Adding bicarbonate (15 mM) induces a rapid onset of flagellar activity, increasing average pathway velocity (VAP) from approximately 30 to >50 μm/s within 2 min. VAP subsequently increases up to 12 min after adding bicarbonate, when it reaches its maximum [184]. Along with VAP, VSL and LIN also increase, while ALH decreases. However, it seems that the effect is not widespread; it only affects specific subpopulations of sperm, as the motility of some cells is almost unaffected by bicarbonate [185]. Based on these results, it can be assumed that before the CASA analysis itself, a 15 min equilibration of spermatozoa in the medium can reduce the influence of kinematic parameters by the effect of HCO_3_^−^ ions.

Currently, most methods of in vitro capacitation and fertilization (IVF) in pigs expose all spermatozoa at the same time to a fixed concentration of 25 mmol/L HCO_3_^−^, providing a static system that bears little resemblance to the in vivo environment, where HCO_3_^−^ concentrations vary dramatically from the cauda epididymis up to the point of fertilization. In addition, current data suggest that the type of sperm movement produced is also dependent on the concentration of HCO_3_^−^, with a concentration of 15 mmol/L HCO_3_^−^ during sperm capacitation causing a more linear movement [186]. In contrast, high concentrations of bicarbonate (i.e., >37 mM) in the capacitation medium in vitro cause strong agglutination in boar spermatozoa [187], which causes sperm clusters with preserved flagellar activity [188]. A high degree of agglutination in the sample can limit CASA motility analysis [125]. A higher and faster agglutination of boar spermatozoa of the head-to-head type occurs mainly in a medium that, in addition to HCO^−^, also contains Ca^2+^ [188,189].

##### Calcium Ions as a Crucial Component for Sperm Motility and Maturation

Calcium (Ca^2+^) ions, which are supplied to the media most often in the form of CaCl_2_.2H_2_O, are essential ions that regulate sperm motility, capacitation, and the acrosomal reaction, i.e., three processes necessary for successful fertilization [190]. The signaling pathways mediated by Ca^2+^ are involved in the modulation of flagellar movement and the development of a unique motility pattern called hyperactivated motility in the sperm of many animal species [36,191]. Unfortunately, in particular, boar spermatozoa incubated in a medium containing a mM concentration of Ca^2+^ tend to agglutinate (head-to-head) [189], and as a result, the analysis of the motility of aggregated spermatozoa using the CASA system becomes difficult [81]. A study by Schmidt and Kamp [81] found that a narrow range of Ca^2+^ concentration in μmol, namely a range of 40 to 70 μmol/L, is sufficient to induce the hyperactivation of boar spermatozoa. Conversely, high concentrations of Ca^2+^ (>100 μmol/L) lead to an almost complete loss of sperm motility, and low concentrations of Ca^2+^ (<20 μmol/L) prevent hyperactivity and reduce sperm motility [81]. Calcium in cells also affects sperm motility by regulating ATP concentration [192].

##### Energy Substrates Prolong Sperm Motility

Mammalian sperm motility is directly dependent on the availability of energy obtained by the hydrolysis of ATP and represents about 70% of its total consumption [193]. It is therefore clear that ATP availability can significantly affect sperm motility. In these cells, ATP concentrations are regulated by a dynamic balance between two primary processes: glycolysis and oxidative phosphorylation (OXPHOS) [194]. In the sperm, ATP synthesized by any of these processes is involved in maintaining progressive motility and is also necessary for changes in the sperm motility pattern known as hyperactivation [192]. Research conducted in the last few years has shown that the exact balance between glycolysis and mitochondrial oxidation pathways varies between species [195]. Although this balance was thought to be incredibly imbalanced in favor of glycolysis in boar sperm (up to 95%) [196], more recent studies suggest that the main energy pathway involved in promoting boar sperm capacitation and hyperactivation is OXPHOS [197]. Even though spermatozoa can also use other sources of energy, including extracellular metabolites such as lactate, pyruvate, citrate, glycerol, or even triglycerides [198], it is generally true that the main source of energy is monosaccharides, especially glucose or fructose [199]. Therefore, their availability in media and diluents is crucial for regulating sperm motility.

##### Effect of Incubation Media and pH on Sperm Motility

The type of media chosen can also affect sperm analysis by the CASA system [200]. Yolk granules are similar in size to sperm and are present in egg yolk diluents, which can adversely affect the assessment of the percentage of motile sperm and, ultimately, the accuracy of the results [201]. In addition, choosing a medium with a higher viscosity can also lead to a decrease in kinematic parameters such as VAP, VCL, and VSL [202].

It is widely known that environmental pH affects sperm motility [203]. In the *Cauda epididymis*, which acts as a repository for functionally mature sperm before ejaculation [204], the pH value is 6.5 [205]. In combination with specific proteins and enzymes, this acidic luminal environment helps maintain the sperm in a state of anabiosis, that is, in a state with reduced metabolic activity and motility [206]. However, during ejaculation, spermatozoa are mixed with accessory gland secretions, increasing the extracellular pH and bicarbonate concentration, and the sperm becomes fully motile [207]. It is reported that boar ejaculate immediately after ejaculation reaches a pH value of 7.4 ± 0.2 [208], while deviations can occur not only within breeds, but also within individual samples from one individual [209]. In the female reproductive tract, spermatozoa are subsequently exposed to an environment whose pH varies from more acidic in the vagina to more basic near the ovaries [210]. Spermatozoa respond to this changing pH by adjusting their motility patterns [211]. In boar spermatozoa, it has been shown that while the percentage of motile sperm and flagellar beat frequency increases with increasing pH from 7 to 8 [212], pH values below 7.2 decrease sperm motility and metabolism [213].

The intracellular pH of sperm is directly related to the pH of the medium [212,214], and the current market offers a variety of commercial diluents and media with different pH values. As sperm glycolytic metabolism lowers intracellular pH, which ultimately reduces cell metabolism and motility, buffers are needed in diluents and media to regulate pH changes [215]. Sperm pH regulation is enabled by three mechanisms, namely the HCO_3_^−^ influx, voltage-gated proton channel (Hv1), and Na^+^/H^+^ exchanger (NHE) mechanisms [216]. Therefore, media typically include simple buffering systems such as bicarbonates or sodium citrate. However, these only have a limited ability to regulate pH, so more complex systems such as TES, HEPES, Mops, or Tris are added to the media and diluents, which can regulate pH over a broader range, even when there are temperature changes [215].

A study by Rivas et al. [211] investigated the effect of pH (7.4, 7.6, 7.8, and 8.0) on sperm motility using commercial doses of boar semen in Androstar Plus diluent. The researchers found that increasing the pH did not affect the proportion of motile sperm but how the sperm moved. While the speed parameters decreased with increasing pH, the values expressing the linearity of the movement increased—sperm movement was slower but more linear. At the same time, the authors demonstrated the presence of three subpopulations whose distribution changed, especially at pH values of 7.8 and 8.0, and whose response to pH changes was different [211]. Changes in pH can significantly affect sperm motility and its analysis by the CASA system.

#### 4.2.5. Beneficial Effects of Higher Viscosity on Sperm Kinematic Parameters

Sperm motility and its kinematic parameters are assessed in an environment that differs considerably from the in vivo environment to which spermatozoa are exposed in the female reproductive tract [217]. One of the properties of these media that differs the most is viscosity. For example, the viscosity of amniotic fluid is more than two orders of magnitude higher than that of water [218]. It is the viscosity of the environment that the sperm encounters that significantly influences their motility [219]. It has been shown that the low viscosity of in vitro media can distort the obtained motility data, especially if its hyperactivation is assessed [220]. To more closely mimic in vivo conditions to improve research and reproduction techniques, studies have begun to appear that incorporate viscosity-increasing compounds into the incubation media and diluents, e.g., acrylamide, powdered plant extracts, hyaluronic acid, carboxymethylcellulose, or methylcellulose [202]. Analysis of the kinematics of the sperm head showed that an increase in viscosity reduces the speed of sperm swimming and the rolling rate of the head (rolling rate), and limits the twisting during their movement due to the increased braking force felt by the cell [221]. In boar sperm, an increase in the viscosity of the medium under non-capacitating conditions has been shown to have a beneficial effect on sperm parameters in the sense of higher linearity and directness of sperm movement, a higher proportion of fast linear sperm and a lower percentage of spermatozoa with slow non-linear movement [202]. A reduction in VCL and an increase in STR and LIN in higher-viscosity media appears to be a general effect observed in various animal species [35,219,220]. It has also been suggested that increased viscosity reduces flagellar wavelength while increasing flagellar curvature, raising the question of the reliability of the BCF parameter, which may not accurately represent flagellar beating based on these observations. A study on human and bull spermatozoa showed that increasing the viscous load on the sperm flagellum triggers the transition from 3D to 2D swimming, regardless of geometric constraints or chemical stimuli. This transition from 3D to a more energy-efficient 2D swimming mode is advantageous at higher viscosities, at which progressive sperm velocity is affected by increased cell resistance. Interestingly, despite the significant increase in drag and higher energy dispersion into the fluid, 2D motile sperm maintain a consistent swimming speed over a wide viscosity range without requiring an increase in metabolic activity [221].

The viscosity of the sample also affects the sperm distribution in the chamber, which is essential for sperm analysis by the CASA system. However, correction factors are currently available based on the time it takes to fill the chamber and are calculated to consider the Segre–Silberberg effect [125,126] (more in Section 4.1).

#### 4.2.6. Temperature Management: Effects on Sperm Motility and Preservation

In many laboratories, one of the basic steps in ejaculate processing is, in addition to dilution, its subsequent cooling to a temperature of 17 °C. At this temperature, seed batches are stored until needed, but usually for a maximum of 4 days. Preserving sperm using a diluent and lowering the temperature slows sperm metabolism, which increases their chance of survival during in vitro preservation [213]. However, boar sperm membranes have a lower concentration of cholesterol. They are thus more sensitive to cold than spermatozoa of other species [222]. When boar sperm is cooled too quickly, lipid phase separation occurs, leading to changes in membrane proteins and membrane permeability, with consequent leakage of cations and enzymes [222,223]. Temperature changes during sperm processing can profoundly impact sperm quality and motility [224]. Two dilution protocols are commonly used in laboratories: (1) one-step dilution, in which boar spermatozoa are diluted isothermally within 30 min of their collection to a final volume with the diluent preheated to approximately 32 °C, and (2) two-step dilution, in which boar spermatozoa are first diluted in a ratio of 1:1 or 1:2 with a diluent preheated to approx. 32 °C, and then the final dilution in a diluent preheated to approx. 32 °C (two-stage isothermal dilution) or in a diluent at room temperature, i.e., 21 to 24 °C (two-step hypothermic dilution) [225]. The most recently established protocol in boars is a two-step hypothermic procedure to facilitate ejaculate processing by bringing semen batches to the required storage temperature more quickly [224].

Two studies investigated the effect of isothermal and hypothermic dilution on boar sperm motility [224,226]. A study by López Rodríguez et al. [224] found that neither total motility, progressive motility, nor any of the kinematic parameters were affected by the chosen protocol, i.e., the dilution temperature. This means, in practical terms, that when a two-step dilution is performed, preheating the diluent for the second dilution step is not required to maintain good sperm quality. This is in contrast to the study by Schulze et al. [226], which showed higher values of total and progressive motility in isothermally diluted spermatozoa. However, the kinematic parameters of motility were not affected by the room temperature of the diluent, as in the aforementioned study.

This method of preserving and maintaining porcine ejaculates has been adopted by numerous laboratories as a standard in investigating the function of boar semen [88]. Generally, boar sperm studies are performed at 38.5 °C, which is the physiological temperature of pigs. To start any experimental protocol, seed lots stored at 17 °C must be pre-warmed up to 38.5 °C [88,227]. However, experimental observations show that motility parameters are modified during this preheating phase in response to the temperature gradient. Exposure to elevated temperature causes an increase in the percentage of boar spermatozoa showing non-progressive motility, characterized by an increase in BCF, ALH, and VCL and a concomitant decrease in LIN. All these temperature-gradient-induced parameter modifications are fully compatible with hyperactive motion. However, once the temperature of the medium is stabilized within approx. 30 min (i.e., the temperature gradient disappears—a minimal increase of 0.03 ± 0.048 °C min^−1^), the sperm return to a more progressive motility pattern. An interesting finding is that temperature-gradient-induced sperm hypermotility is independent of extracellular Ca^2+^ and PKA activity. Moreover, the hyperactive sperm population induced by the temperature gradient differs in its parameters from the hyperactive population caused by the calcium ionophore [88].

Similar results were also demonstrated in human spermatozoa, in which temperature changes cause an increase in hyperactive motility, thanks to which sperm can redirect their trajectories to the warmest places [228,229]. Moreover, these changes in motility patterns appear to be rapid and transient and occur within 3–10 min of exposure to the new temperature. After that, the motility parameters partially return to their original values [228].

It is therefore clear that temperature change can significantly affect sperm motility and kinematic parameters, as analyzed by the CASA system. It seems that for the study of motility and its characteristics, it is appropriate to use native ejaculate instead of refrigerated semen doses. This step can minimize the temperature gradient effect that occurs when heating chilled seed batches to a temperature of 38.5 °C, thanks to which we can obtain more objective data.

#### 4.2.7. Breed as an Important Factor Behind Variability in CASA Results

The difference in sperm motility and kinematic parameters is observed not only between animal species, but also between breeds of the same species, as was demonstrated in boars [103] as well as dogs [104] and stallions [230]. One explanation for this phenomenon appears to be differences in sperm shape and size between breeds [231], as even slight differences in head size and morphology can lead to significant changes in sperm hydrodynamics that will affect sperm motility [232]. This needs to be taken into account not only when preforming evaluation with the CASA system, but also when comparing studies conducted on one animal species.

### 4.3. Species-Specific Analysis Settings and Standardization

The accuracy and sensitivity of each output measurement also depend on the software system settings and the correct definition of criteria associated with species-specific sperm characteristics. There are various components of CASA systems, such as cell size, contrast, cell intensity (pixels), illumination, minimum observation time, etc., whose settings vary from animal species to animal and are therefore crucial for motility analysis. Although various CASA systems with updated parameter settings and modifications for multi-species use are currently available on the market [200], there are differences in the settings used across laboratories within the same species, even when using the same device model. The study by Baqir et al. [233] demonstrates how inappropriate or inconsistent CASA system setups significantly affect the analysis outcomes for sperm kinematics, particularly in species without tailored CASA configurations. Working with Arabian leopard semen, the researchers found high variability in motility and progressive motility parameters across different CASA species settings. For instance, motility varied from 37.9% using stallion settings to 88.3% with porcine settings, illustrating the critical impact of species-specific configurations. O’Meara et al. [97] evaluated the effect of different CASA IVOS II settings on bovine sperm motility, concentration, and morphology. In this study, it was evident that different results were obtained when changing the threshold settings, especially for progressive motility. Changing from low to high thresholds resulted in a 20% and 40% difference in the results obtained for sperm straightness and average trajectory velocity, respectively. CASA IVOS II has also been utilized in the study by Jorge-Neto et al. [98] for the optimization of a CASA setup for the analysis of elasmobranch spermatozoa. This study emphasizes the importance of the working temperature taking into account whether the species is ectothermic or endothermic. The Live Configuration tool in the CASA system allowed for species-specific adjustments, ensuring accurate sperm head detection while minimizing artifacts such as sloughed cells or microorganisms. For elasmobranchs, adjustments such as modifying “Head Brightness Min” scores ensure the reliable tagging of sperm heads. It is therefore clear that the effectiveness of CASA depends on tailored standardization for the species studied, as different settings can significantly affect the results. This is also related to the proper maintenance of CASA systems, including the regular verification of phase contrast adjustments, which is essential for preventing analytical errors [98]. It is the variability of CASA results, combined with the absence of standardized settings, that reduces the reliability of sperm assessment and highlights how inappropriate parameter thresholds compromise data integrity. The practical consequences of in vivo fertility, especially in bulls [234] and boars [235,236], are usually derived from the data obtained by the CASA system. The findings of a recent study by Savić et al. [236] suggest that while total motility is a useful predictor, parameters such as progressive motility and sperm dose count play a more significant role in determining fertility. Higher sperm dose counts were associated with improved fertility rates up to a threshold, beyond which no significant benefits were observed. Fertility outcomes were best predicted when considering both motility and dose count together. Boars with high progressive motility required fewer sperm per insemination to achieve comparable fertility rates to those with lower motility.

Recently, studies strengthening the link between sperm motility parameters measured by CASA systems and their practical implications for in vivo fertility outcomes further advocate the use, development, and standardization of species-specific CASA configurations. An example of a procedure for creating a standardized setup and gold standard protocol for semen evaluation can be found in studies by de Araújo et al. [237] and Jorge-Neto et al. [98].

### 4.4. Frame Rate—A Key Variable Influencing CASA Evaluation

One of the most important settings of any CASA-Mot system and, therefore, the most important variable is the FR [93], which indicates the number of frames displayed per second [73] and thus allows the reconstruction of the true sperm trajectory [105]. FR depends mainly on the quality of the video camera and the imaging standard used [73], so the FR used was until recently limited to 16 to 60 fps (frames per second) [111,238]. With advances in technology, much more efficient cameras that allow working with higher FRs of up to 500 fps have reached the market; however, according to Gacem et al. [239], there is no software that can analyze the huge amount of data generated with an FR higher than 250 fps. Another problem that arises is that high FR values result in significant changes in the values of some sperm kinematic parameters [106]. This is because FR and video length affect the distance a sperm can move between successive frames [240]. This has a direct effect on the estimated trajectory and its deviations for each sperm cell [4]. It has been shown that using fps below 50, it is not possible to obtain trajectory characteristics that occur at intervals shorter than the time elapsed between frames [106,241]. Because of this aspect, information about the “true” trajectory may not be obtained, which may further influence the results of kinematic parameters of sperm motility, especially VCL, BCF, and VAP [111,242]. Even though the FR value does not have an obvious effect on the overall motility [106,242], it is clear that the movement trajectory changes with the FR value, as a larger FR indicates a trajectory more similar to the actual course of the cell [243]. The results across species confirm the observation of increasing speed parameters with increasing FR, which is particularly important for the assessment of hyperactivation. While the most sensitive parameter is VCL, which is calculated using the reference point positions in each frame, the least sensitive parameter is VSL, which is given purely by the connection between the first and last point of the track [105]. According to the study by Castellini et al. [242], LIN (VSL/VCL) decreases with increasing FRs due to the increase in VCL, since VSL is completely independent of the FR value and remains the same. Together with LIN, ALH also decreases, which the authors explain by the fact that a higher frequency of imaging reduces the distance of the deviation of the sperm head from the average path. A substantial increase in BCF continues to be associated with this trend [242]. Based on these findings, it is clear that one cannot simply compare data from studies using different FRs. Although the 60 fps CASA system in principle meets the requirements for analyzing sperm dynamics in humans and other mammals [74], the effort to determine the optimal frequency (FRO) for specific applications depends on the animal species as well as the type and depth of chambers [239].

A study by Valverde et al. [106] compared six FR settings (25, 50, 75, 100, 150, and 200 fps) on boar spermatozoa using the ISAS^®^v1 CASA-Mot system and confirmed through a regression model that the parameter most sensitive to the FR value is VCL, for which there was a non-linear correlation that exhibited an asymptotic level at 212 fps. This value, defined as the optimal frame rate (OFR), represents the threshold at which multiple captures lead to the same sperm path. Thus, for sperm that exhibit non-linear trajectories, multiple captures will be needed to define the correct track [239]. This explains the differences obtained when calculating the OFR for salmon (250 fps) [92], bull (256 fps) [244], donkey (278 fps) [239], or ram and rabbit sperm (302 fps) [242]. Boar spermatozoa exhibit a lower OFR value when compared to other species, which suggests that their motility is not as variable and/or the sperm are not as fast [106]. However, when determining the OFR, it is also necessary to take into account the type and depth of the chamber used, as demonstrated by the study by Gacem et al. [239].

In boar spermatozoa, a clear decrease in LIN and STR parameters was observed with increasing FR value, as a result of VSL being constant and a higher increase in VCL than VAP [106,235], which is a general trend observed in spermatozoa of many animal species (reviewed by Barquero et al. [235]). An increase in the values of the BCF parameter, which is sensitive to changes in the direction of movement of the head as a result of whiplash, was also clear. However, inconsistent results were observed for WOB and ALH parameters within these studies. While Valverde et al. [106] observed a decrease in WOB with an increasing FR value up to the maximum tested FR value, Barquero et al. [235] observed a decrease in the WOB value only up to 100 fps, after which there were no relevant changes for values up to 200 fps. The complete opposite results were found for the ALH parameter. Barquero et al. [245] reported that ALH values tended to decrease with increasing frame rate, while this tendency was also described in other studies on sperm from other animal species [242]. In contrast, the study of Valverde et al. [106] reported that ALH values did not change significantly from 50 fps, in fact exhibiting a slight increase at higher FRs. It is generally known that ALH is the most sensitive parameter to algorithms that are specific to individual manufacturers [246]. However, the same CASA-Mot system ISAS^®^v1, the same sample volume in the same ISAS^®^D4C20 chamber, and even the same breed at the same age were used for analysis in the two studies.

Depending on the frame rate used, the distribution of sperm subpopulations also differs [106,235], i.e., cell groups with different movement and kinematic patterns [247,248]. The most recent studies located the same four sperm subpopulations in the ejaculate; however, in both studies, the sperm distribution within the subpopulations and also the values of individual kinematic parameters characterizing specific subpopulations differed significantly between analyses with different FR settings [106,235]. However, it is important to note that the source of deviations can also be different algorithms within different CASA-Mot brands; therefore, the kinematic values for each subpopulation may differ, even with the same FR setting and chamber used [95].

Based on the available studies, it is clear that frame rate is a factor that significantly affects sperm kinematic parameters and therefore needs to be defined to standardize CASA analyses between laboratories and avoid introducing sources of external variation [245]. It appears that 50 fps for 0.5 s can be used as a reference setting for the commercial evaluation of total boar sperm motility [104]. For studies based only on progressive motility, Valverde et al. [106] use a 100 fps FR for 0.5 s. However, the study and evaluation of kinematic parameters require higher-resolution cameras, thanks to which a higher FR can be used to achieve greater accuracy in reconstructing the true trajectory of the cell [245]. If computing memory is not a limiting factor, it is preferable to shoot as long as possible at a high number of frames per second, ideally for 2 s at a min. of 200 fps [104,106].

### 4.5. Reproductive Seasonality and Its Effects on Overall Boar Sperm Quality

The quality of domestic animal spermatozoa is influenced by many factors, such as individual variability, age, breed, season, nutrition, frequency of semen collection, and seminal plasma composition [249,250]. Specifically, in boars, these factors can cause changes in sperm production over the year by approximately 25 to 30% [251]. It has been shown that reproductive seasonality is one of the most important aspects that influence the quality of boar ejaculate, including its motility [249].

Reproductive seasonality in the domestic pig (*Sus scrofa*) is a trait inherited from its ancestor, the European wild boar (*Sus scrofa ferus*), whose breeding activity occurs in late winter and early spring, while it is subdued in summer and early autumn [249]. The effect of this factor on sperm motility has been the subject of several studies. Fraser et al. [252] demonstrated a significant effect of seasonality on total and progressive motility and velocity parameters (VAP, VSL, VCL), with higher values obtained in winter. This correlates with the results of the study by Ibanescu et al. [253], which showed that in the winter period, higher values of total and progressive motility, speed (VAP, VCL, VSL), ALH, and BCF are recorded in spermatozoa, while in the summer period, spermatozoa exhibit higher values of STR, LIN, and WOB [253]. The parameter most responsive to annual changes seems to be VCL [95]. Also on the basis of other studies [248,254,255], it can be said that the total number of butterfly sperm drops (up to 20%) [255] in the summer during long days, when the pattern of sperm swimming is also affected—they move at a lower speed, but more regularly, which can be correlated with a more efficient consumption of energy resources. Conversely, winter improves sperm metabolism, increasing the percentage of motile spermatozoa and their speed [252].

However, it has been shown that the time of year of collection has a great influence on the distribution of sperm subpopulations in boar ejaculate [95]. The fast and linear sperm subgroup, which is considered by some authors to have the highest fertilization potential [256,257], was best represented in the winter period and poorly represented in the autumn and summer months [95]. Thus, seasonal dynamics in kinematic parameters may be due to changes in the proportion of spermatozoa assigned to different subpopulations, rather than an overall increase/decrease in values for all ejaculated spermatozoa. This would mean that the summer period only affects a certain percentage of spermatozoa, whose reduced speed results in a decrease in the overall average speed [95].

It is clear that reproductive seasonality must be taken into account as a factor that can significantly affect the data obtained by the CASA system. According to some studies, when analyzing basic sperm parameters, seasonality has an even stronger effect than age [250].

## 5. Conclusions

CASA is a tool for more objective and accurate analysis of sperm motility, widely used in research laboratories and insemination center laboratories. However, the objectivity of the obtained data and outputs from CASA is affected by a number of factors that influence not only the analysis itself, but also the sample preparation and finally statistical processing of the data and relevant interpretation. Without a standard operational procedure for the CASA of spermatozoa, it is somewhat difficult to perform interlaboratory comparisons either in clinical practice or basic research. Validation of these factors, regular staff training and the creation of a standardized procedure could provide assurance of quality CASA analysis and reduce possible deviations of the obtained data even within laboratories.

## Figures and Tables

**Table 1:** Values for hyperactivated boar sperm reported by various studies.

Study	FR	ALH (μm)	VCL (μm/s)	VAP (μm/s)	VSL (μm/s)	LIN (%)	STR (%)	WOB (%)	BCF (Hz)
Schmidt and Kamp [81]	50	8.0 ± 1.8 > 3.5	151.6 ± 33.5 > 97	88.1 ± 18.6	16.8 ± 9.1	11.8 ± 7.3 < 32	20.8 ± 13.6	58.6 ± 6.3 < 71	8.0 ± 2.1
Mircu et al. [86]	NA	±7.0	±150	NA	NA	<50	NA	NA	NA
Broekhuijse et al. [87]	60	7.3 ± 1.3	175.2 ± 37.3	95.1 ± 20.5	68.5 ± 18.4	NA	NA	NA	39.3 ± 2.8
Martin-Hidalgo et al. [88]	25	7.0 ± 0.1	146.1 ± 3.1	61.4 ± 1.8	20.3 ± 0.6	14.1 ± 0.4	34.1 ± 0.9	41.9 ± 1.4	6.1 ± 0.4

FR, frame rate; ALH, amplitude of lateral head displacement; VCL, curvilinear velocity; VAP, average path velocity; VSL, straight-line velocity; LIN, linearity; STR, straightness; WOB, wobble; BCF, beat cross frequency (BCF); NA, not analyzed.

## Data Availability

Not applicable.
